# Retrospective Analysis Using Pharmacokinetic/Pharmacodynamic Modeling and Simulation Offers Improvements in Efficiency of the Design of Volunteer Infection Studies for Antimalarial Drug Development

**DOI:** 10.1111/cts.12934

**Published:** 2020-12-16

**Authors:** Kayla Ann Andrews, Joel S. Owen, James McCarthy, David Wesche, Nathalie Gobeau, Thaddeus H. Grasela, Jörg J. Möhrle

**Affiliations:** ^1^ Cognigen Corporation a SimulationsPlus Company Buffalo New York USA; ^2^ Department of Pharmaceutical Sciences State University of New York at Buffalo Buffalo New York USA; ^3^ The Royal Melbourne Hospital The University of Melbourne at the Doherty Institute Melbourne Australia; ^4^ Certara Strategic Consulting Princeton New Jersey USA; ^5^ Medicines for Malaria Venture Geneva Switzerland

## Abstract

Volunteer infection studies using the induced blood stage malaria (IBSM) model have been shown to facilitate antimalarial drug development. Such studies have traditionally been undertaken in single‐dose cohorts, as many as necessary to obtain the dose‐response relationship. To enhance ethical and logistic aspects of such studies, and to reduce the number of cohorts needed to establish the dose‐response relationship, we undertook a retrospective *in silico* analysis of previously accrued data to improve study design. A pharmacokinetic (PK)/pharmacodynamic (PD) model was developed from initial fictive‐cohort data for OZ439 (mixing the data of the three single‐dose cohorts as: *n* = 2 on 100 mg, 2 on 200 mg, and 4 on 500 mg). A three‐compartment model described OZ439 PKs. Net growth of parasites was modeled using a Gompertz function and drug‐induced parasite death using a Hill function. Parameter estimates for the PK and PD models were comparable for the multidose single‐cohort vs. the pooled analysis of all cohorts. Simulations based on the multidose single‐cohort design described the complete data from the original IBSM study. The novel design allows for the ascertainment of the PK/PD relationship early in the study, providing a basis for rational dose selection for subsequent cohorts and studies.

**Study Highlights**

**WHAT IS THE CURRENT KNOWLEDGE ON THE TOPIC?**

☑ Volunteer infection studies are routinely used in antimalarial drug development to generate early pharmacokinetic/pharmacodynamic data for compounds.

**WHAT QUESTION DID THIS STUDY ADDRESS?**

☑ Can *in silico* analyses be used to suggest improvements to volunteer infection study designs?

**WHAT DOES THIS STUDY ADD TO OUR KNOWLEDGE?**

☑ Multiple dose adaptive trial designs can potentially reduce the number of cohorts needed to establish the dose‐response relationship in volunteer infection studies.

**HOW MIGHT THIS CHANGE CLINICAL PHARMACOLOGY OR TRANSLATIONAL SCIENCE?**

☑ Real time data analyses can be used to recommend doses for adaptive volunteer infection studies.

Volunteer infection studies using the induced blood stage malaria (IBSM) model have been recognized as a valuable system for defining the key pharmacokinetic (PK) and pharmacodynamic (PD) relationships for dose selection in antimalarial drug development.^1^, ^2^, ^3^, ^4^, ^5^, ^6^, ^7^ In such studies, healthy volunteers are inoculated intravenously with a given quantity (with small variability) of *Plasmodium*‐infected red cells. Parasitemia is then followed by quantitative polymerase chain reaction until a prespecified treatment threshold is reached when the test drug is administered. Parasite and drug concentrations are then measured. These studies are conducted prior to phase II dose‐response (D‐R) trials and can be included in an integrated first‐in‐human study protocol, or after completion of the first‐in‐human PK and safety study. IBSM studies have been typically designed as flexible multiple cohort studies where each volunteer of one cohort receives a single dose of the same amount of drug (“single dose per cohort”).^2^, ^3^, ^4^, ^5^ After each cohort, a decision is made to stop or to add a cohort to test a lower or higher dose based on the response observed in the previous cohorts.

For the multiple single‐dose‐per‐cohort design, the starting dose is typically selected based on safety and PK information from a phase I single ascending dose (SAD) study and, more recently, on preclinical data from a severe combined immunodeficient mouse model, with the dose selected on the basis of being best able to inform the D‐R relationship, rather than aiming for cure. This approach, where a single dose is tested in all subjects of the initial cohort, risks missing the dose likely to be most informative for defining the PK/PD relationship.

An alternative approach is to spread a range of doses across a smaller number of subjects within the initial cohort and use PK/PD models developed based on data from this cohort to support dose selections of subsequent cohorts and studies. Using data from a previous study,^2^ we undertook an *in silico* investigation of such an adaptive study design, aiming to reduce the number of subjects exposed to inefficacious doses, and to establish a D‐R relationship. This multiple‐dose‐groups‐per‐cohort design, referred to as the “2‐2‐4” design, is contrasted with the already implemented study design depicted in **Figure** **1**.

**Figure 1:**
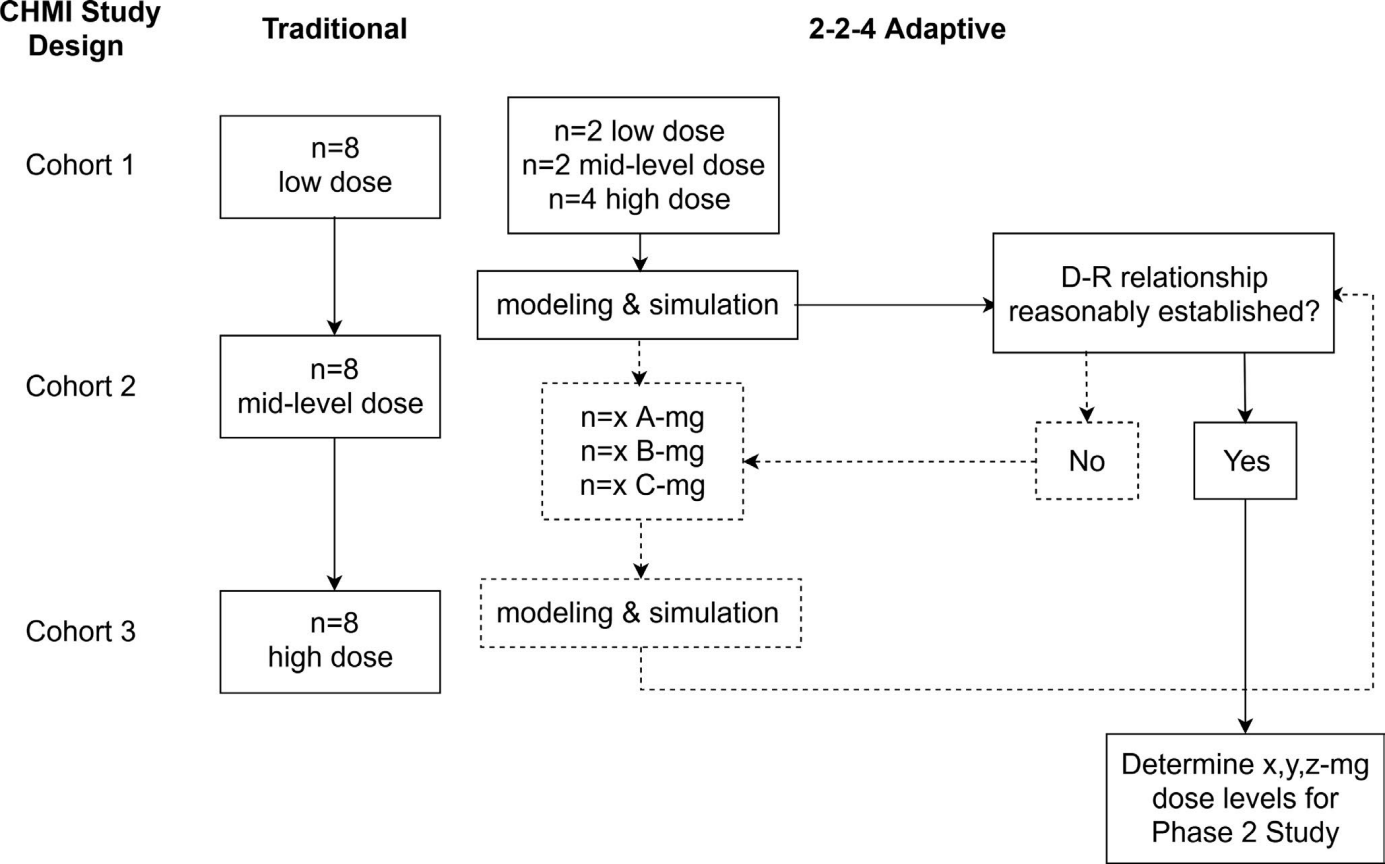
Comparison of standard and adaptive designs of IBSM studies. A/B/C, dose levels to be selected during the progress of the study based on pharmacokinetic/pharmacodynamic results of the initial cohort; CHMI, controlled human malaria infection; D‐R, dose‐response; IBSM, induced blood stage malaria infection; n, number of subjects at each dose.

The objectives of this retrospective analysis were to: (i) compare PK/PD parameter estimates from the initial cohort of the 2‐2‐4 study design with the prior results from the data of the full study and (ii) propose a preliminary workflow to establish D‐R early in an IBSM study, and use modeling and simulation (M&S) to support dose selections for subsequent cohorts and later phase clinical trials.

## METHODS

### Hypothetical 2‐2‐4 cohort creation

The *P. falciparum* IBSM study that served as a basis for this work was conducted to investigate the efficacy of a drug, OZ439, in three sequential ascending‐dose cohorts. The study was approved by the QIMR Human Research Ethics Committee and registered on anzctr.org.au (registration number ACTRN12612000814875). All participating volunteers provided written consent. Eight subjects in each cohort received a single dose administration of 100, 200, or 500 mg of OZ439, 168 hours after inoculation with *P. falciparum*. The results of this study have been published elsewhere.^2^ The 100 mg dose cohort did not result in an observable drug effect, and parasitemia continued to rise in all subjects; therefore, all subjects required rescue medication after 48 hours. The second dose level exhibited some pharmacologic effect, and the 500 mg dose level exhibited the greatest effect.

Data from 8 of the 24 subjects in this study trial were randomly selected to build a cohort consisting of 2 subjects from the 100 mg dose group, 2 subjects from the 200 mg dose group, and 4 subjects from the 500 mg dose group. Because IBSM studies have typically included 8 subjects per cohort, and the increased drug efficacy with increasing doses was known, the 2‐2‐4 design was selected for this proof‐of‐concept work. To obviate any gender‐related bias, half of the subjects were men, and half were women, at each dose level. No outlier samples or subjects were excluded from the analyses.

### Software

All exploratory data analyses and presentations of data were performed using SAS version 9.4 (SAS Institute, 2013) and KIWI Version 2.0 (Cognigen, 2018). Population modeling and stochastic simulations were performed using the computer program NONMEM, version 7.3.0 (ICON Development Solutions, 2013). NONMEM analyses were performed on an Intel cluster with the Linux operating system. The first‐order conditional estimation method with interaction was used to approximate the maximum likelihood estimation of the nonlinear mixed effects models used to characterize the PK and PD data.

### Model evaluation and diagnostics

Prior to model development, an exploratory data analysis was performed. Summary statistics of subject characteristics were calculated (**Table** **S1**). Mean parameter estimates and their standard errors, the minimum value of the objective function (for hierarchical models), magnitude of residual variability and magnitude of interindividual variability (IIV) were compared between models. A series of goodness‐of‐fit diagnostic plots, as well as an overlay plot of individual observed data with population predictions and individual predictions, were generated and evaluated for each model.

### Pharmacokinetic model development

In accordance with the M1 method, three samples that were below the lower limit of quantification (LLOQ; 0.1 ng/mL) were removed prior to analysis.^8^ The data from the 2‐2‐4 cohort were used to develop a PK model; two‐compartment and three‐compartment models were tested with allometric functions of body weight on clearance and volume parameters. IIV on all PK parameters was evaluated by testing various combinations of random effect terms on different parameters. The PKs and PDs were modeled sequentially whereby each subject’s empirical Bayesian estimates of PK parameters were used to fit the PD data.^9^

### Pharmacodynamic model development

The quantitative polymerase chain reaction parasitemia data included the first parasite sample observed postinoculation and through treatment. Of the 132 parasite samples included in the dataset, 20 samples were below the LLOQ (10 parasites/mL); these were set to half the LLOQ and were retained in the dataset in accordance with the M5 method.^8^ During model development, the M1, M3, and the M5 methods were tested; the M5 method yielded the best model fit. Parasite growth and net parasite growth were evaluated with log‐linear, logistic, and Gompertz‐type functions. Drug effect was evaluated with a maximum pharmacologic effect model, as well as with a maximum pharmacologic effect model with an indirect response component.

### Model comparison: 2‐2‐4 vs. full study

Using the final PK/PD model developed from the 2‐2‐4 cohort, the data from the full IBSM study were fit and the PD model parameters were re‐estimated. Additionally, a prediction‐corrected visual predictive check (pcVPC) of the final PK/PD model generated from the 2‐2‐4 cohort was plotted with the observed data from the full study.

## RESULTS

### Pharmacokinetic model

OZ439 concentration vs. time plots showed multiple log‐linear phases in the decline in concentrations (**Figure** **S1**). A plot of the dose‐normalized observed concentration data vs. time demonstrated a lack of dose proportionality (**Figure** **S2**). The final PK model included three distribution compartments with first‐order elimination from the central compartment. Absorption was modeled as a duration of zero‐order input into the gut compartment and first‐order absorption into the central compartment. Relative bioavailability was estimated as a proportional shift for doses ≥ 200 mg with the 100 mg dose group as the reference. The final PK model included an allometric function of weight on clearance with a fixed exponent of 0.75. IIV was estimated on clearance and volume of the second peripheral compartment. All fixed‐effect parameter estimates were reasonable and estimated with good precision (**Table** **1**). No trends or patterns were observed in the goodness‐of‐fit diagnostic plots (**Figure** **S3**), and the population and individual model predictions overlaid with the observed data demonstrated good agreement (**Figure** **S4**).

**Table 1:** Parameter estimates and standard errors for OZ439 final pharmacokinetic model for 2‐2‐4 cohort

Parameter	Final parameter estimate		IIV/residual variability	
	Typical value	%RSE	Magnitude	%RSE
*K*_a_: absorption rate, 1/h	0.271	10.6	NE	NA
CL: clearance, L/h	86.9	11.6	22.9 %CV	54.1
CL: exponent of weight on CL	0.750	FIXED		
V2: volume of central compartment, L	72.8	22.2	NE	NA
Q3: intercompartmental clearance, central and first peripheral, L/h	12.8	13.4	NE	NA
V3: volume of first peripheral compartment, L	2530	27.2	NE	NA
Q4: intercompartmental clearance, central and second peripheral, L/h	27.6	19.7	NE	NA
V4: volume of second peripheral compartment, L	280	18.5	52.0 %CV	67.2
D1: duration of zero‐order input, hours	3.00	10.0	NE	NA
F1: proportional shift in relative bioavailability for 200 and 500 mg	0.694	27.1	NE	NA
RV: residual variability	0.0747	14.7	27.3 %CV	NA

Minimum value of the objective function = 697.631.

%CV, coefficient of variation expressed as a percent; %RSE, relative standard error expressed as a percent; IIV, interindividual variability; NA, not applicable; NE, not estimated.

The eta shrinkage was < 3% for CL and V4.

The condition number (ratio of the largest to smallest eigenvalue) was 54.9.

### Pharmacodynamic model

**Figure 2:**
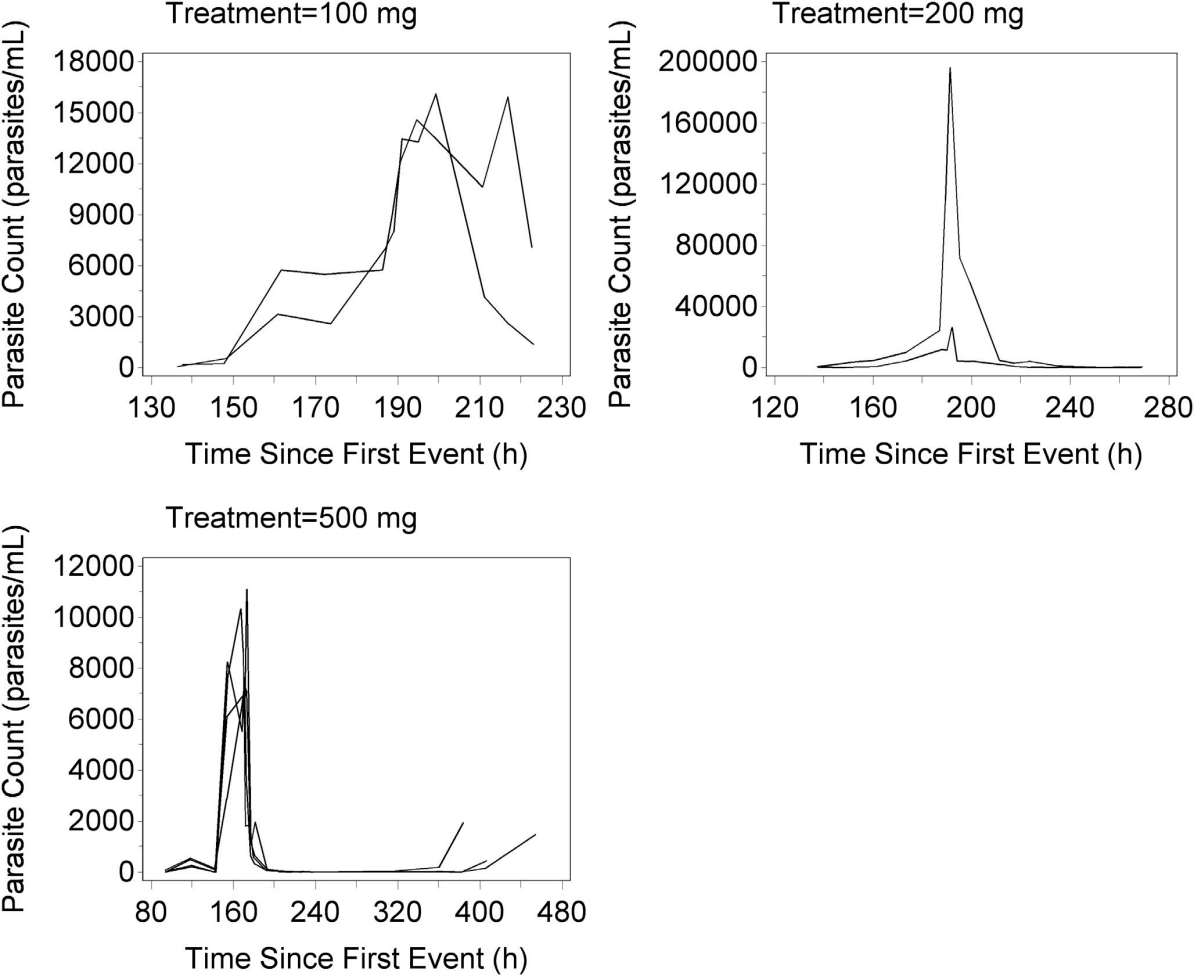
Spaghetti plots of parasite profiles for OZ439 for 2‐2‐4 cohort.

Spaghetti plots of parasite count vs. time by dose group showed great variation in parasite count (**Figure** **2**). Parasite counts and drug concentrations vs. time were overlaid to approximate the initial estimate for concentration at which 50% of maximum rate of parasite death occurs (EC_50_), and values between 5 and 20 ng/mL were tested as initial estimates (**Figure** **S5** **a,b**).

**Figure 3:**
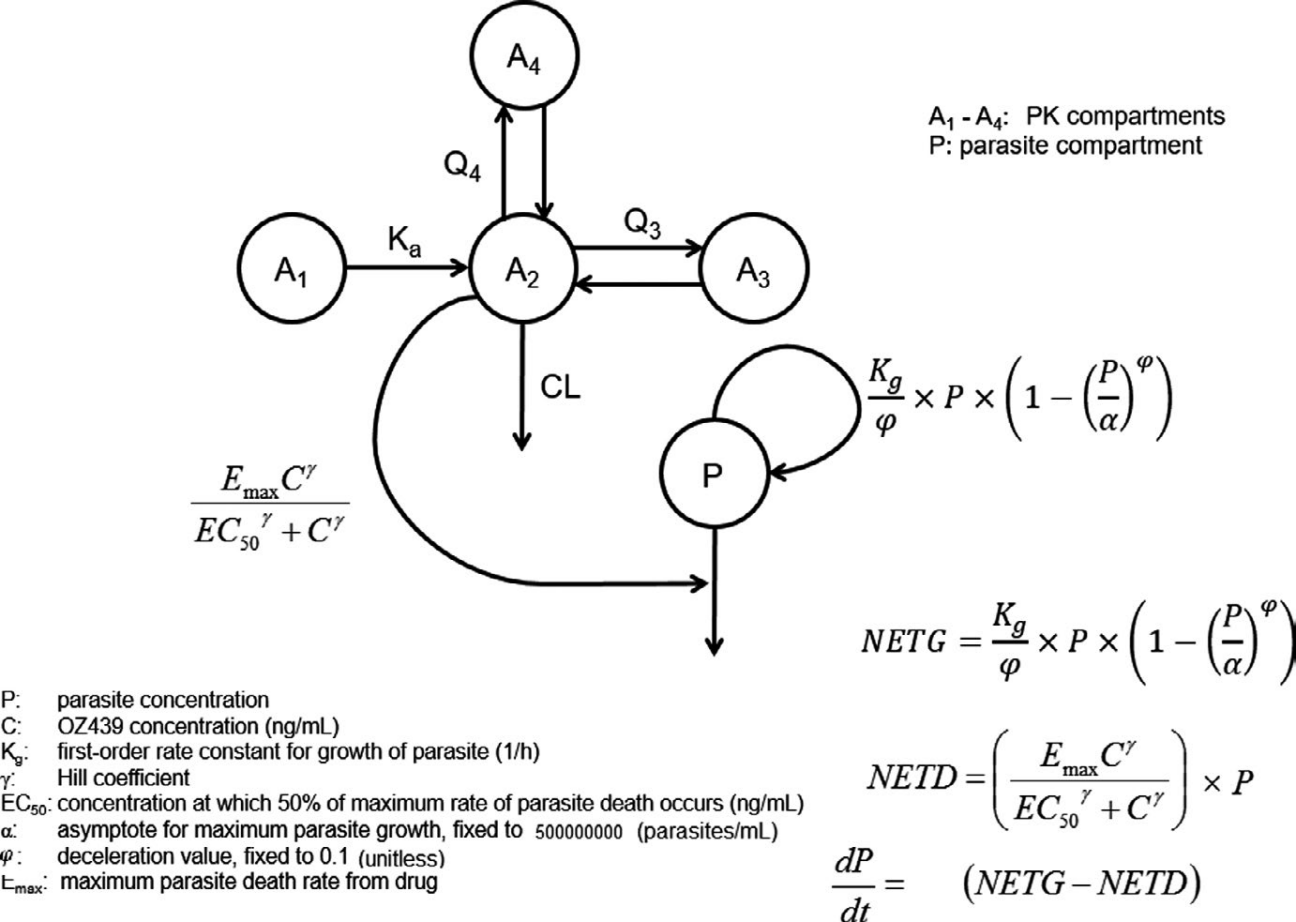
OZ439 pharmacokinetic/pharmacodynamic structural model of 2‐2‐4 data. NETD, parasite death rate due to drug effect; NETG, net parasite growth rate; PK, pharmacokinetic.

The final PK/PD model using initial cohort data and its equations are shown in **Figure** **3**. The final PD model had net growth expressed as a Gompertz‐type function and drug effect characterized with a Hill function for pharmacologic effect. To allow flexibility in the model, a Hill coefficient (gamma) was used which represents the steepness parameter for the concentration‐response relationship; however, this parameter was not estimable with the data from the 2‐2‐4 cohort. During model development, gamma values between 1 and 4 were tested; the value of 1 ultimately provided the best model fit. Residual variability was best described by a log‐error model. Given the many‐fold range of the parasite count data, the selection of a log error model was anticipated. The final model had reasonable parameter estimates with acceptable precision on fixed effect terms (**Table** **2**).

**Table 2:** Parameter estimates and SEs for final OZ439 pharmacodynamic model for 2‐2‐4 cohort

Parameter	Final parameter estimate		IIV/residual variability	
	Typical value	%RSE	Magnitude	%RSE
*K*_g_: first‐order growth rate of parasite, 1/h	0.00990^a^	25.7	NE	NA
EC_50_: concentration of OZ439 at 50% of maximum parasite death, ng/mL	8.22	60.6	40.2 %CV	479
Gamma: steepness parameter for concentration response, unitless	1.00	FIXED	NE	NA
E_max_: maximum parasite death rate from drug, 1/h	0.183^a^	40.8	16.7 %CV	354
Parasite count at baseline, parasites/mL	1800/5000	FIXED	163 %CV	474
Pharmacokinetic residual variability	0.0747	FIXED	27.3 %CV	NA
Pharmacodynamic residual variability SD, log unit	0.353	24.9	0.594 SD	NA

Minimum value of the objective function = 668.3.

%CV, coefficient of variation expressed as a percent; %RSE, relative standard error expressed as a percent; EC_50_, half‐maximal effective concentration; E_max_, maximum effect; IIV, interindividual variability; NA, not applicable; NE, not estimated.

The eta shrinkage was < 36% for *K*_g_, EC_50_, and E_max_.

The condition number (ratio of the largest to smallest eigenvalue) was 179.7.

^a^ These parameter estimates were found to be highly correlated (*r* ^2^ ≥ 0.810).

Overall, the model showed a good fit between the observed data and the individual model predictions; plots of individual weighted residuals vs. individual predictions did not show any biases and plots of the conditional weighted residuals vs. time showed stationarity (**Figure** **S6**). Baseline parasite count was fixed to be the inoculum parasite quantity (1,800 parasites injected) divided by 5 L, the approximate volume of extracellular fluid volume. The model included a random effect for IIV in baseline to account for error in the assumed initial baseline parasite concentrations. There was large IIV on baseline parasite count, which was expected due to the small number of subjects as well as the physiological differences in body size between subjects that may cause parasites to grow at varying rates.^10^ The IIV baseline parameter was more precisely estimated in the full study data (percent relative standard error (%RSE) = 65%), whereas being poorly estimated in the 8‐subject subset of the present analysis (%RSE = 474%). Plots of the observed data vs. individual and population predictions for each subject demonstrated the model’s ability to capture recrudescence accurately (**Figure** **S7**).

Given that the goal of the initial cohort is to define a D‐R relationship for planning future cohorts, model evaluation included a comparison of the fit of the model developed from the 2‐2‐4 cohort to the full study data as well as pcVPCs. A pcVPC of the model developed from the IBSM study overlaid with the observed data from the full study shows most of the observed data falls within the 80% confidence interval of the 2‐2‐4 cohort model predictions (**Figure** **S8**). **Table** **3** shows a comparison of the parameter estimates generated from fitting the model to the full study data vs. the estimates from the 2‐2‐4 cohort; confidence intervals about the parameters as well as RSEs are found in **Tables** **S2–S4**. Of note, the SEs of the PD parameters from the model developed from the 2‐2‐4 exercise were not unreasonable, but it is anticipated that as more cohorts complete, these SEs will become smaller, yielding even more confidence in the PD model parameters. When the 2‐2‐4 model was fit to the full study data, the values for first‐order net growth of parasite (*K*_g_) and maximum drug effect (E_max_) were very similar (**Table** **3**), although %RSE values were smaller in the case of the full study data. It is expected that these numbers will differ because, theoretically, as the number of subjects increase, the accuracy of the parameter estimates improves. The EC_50_ value obtained from the 2‐2‐4 cohort (8.22 ng/mL) was lower than that obtained from the full dataset (21.2 ng/mL), however, the estimate from the full dataset is only slightly higher than the upper bound of the 95% confidence interval from the 2‐2‐4 cohort. Given the total range of concentrations from the study, these values are relatively close. Of note, the %RSE of the EC_50_ value increased by 20% when the model was estimated with the data from the full IBSM dataset.

**Table 3:** Pharmacodynamic model estimate comparison

Parameter	Full study	2‐2‐4 cohort (%RSE)
*K*_g_: first‐order growth rate of parasite, 1/h	0.0092 (7.35)	0.0099 (25.7)
EC_50_: concentration of OZ439 at 50% of maximum parasite death, ng/mL	21.2 (82.8)	8.22 (60.6)
Gamma: steepness parameter for dose response, unitless	1 (FIXED)	1 (FIXED)
E_max_: maximum parasite death rate from drug, 1/h	0.163 (12)	0.183 (40.8)
IIV on baseline parasite count	3.29 (64.9)	2.65 (474)
IIV on EC_50_	5.35 (91)	0.161 (479)
IIV on E_max_	0.0627 (134)	0.0278 (354)
Pharmacodynamic residual variability SD (log unit)	0.271 (9.59)	0.353 (24.9)

%RSE, relative standard error expressed as a percent; EC_50_, half‐maximal effective concentration; E_max_, maximum effect; IIV, interindividual variability.

## DISCUSSION

Adaptive approaches for clinical trials—whereby particular design details of the trial change as the study progresses—have been discussed extensively in the literature^11^, ^12^; and regulatory agencies have also recognized their value and efficiency.^13^ Our work suggests that the design of such volunteer infection studies may be optimized using an initial cohort made up of three dose levels, paired with M&S to potentially reduce the number of subjects required to characterize the D‐R relationship. The 2‐2‐4 design allowed for the characterization of a D‐R relationship after administering drug to only eight subjects in one cohort. The inclusion of three doses in the first cohort allows for early estimation of the key PD parameters (e.g., E_max_ and EC_50_) using data with a wider dynamic range, which would not have been possible using the cohort 1 data of the study as it was run (i.e., using the data from eight subjects receiving 100 mg OZ439). The D‐R relationship established after the first cohort may not be sufficient to estimate the dose needed to cure subjects, but it could be used to select appropriately the most informative doses to be tested in the next cohorts to help refine the D‐R relationship.

The 2‐2‐4 design was arbitrary in this exercise as compared with a 3‐3‐3 design, and the choice of the number of doses, number of subjects per dose level, and selection of doses will depend on the compound to achieve an estimation of minimal dose efficacious in subjects within the expected confidence. Additionally, symmetry arguments would favor a 2‐4‐2 design; the proposal of 2‐2‐4 implies that the highest dose tested in the volunteer infection study is the highest feasible dose, which may not be the case.

This proof‐of‐concept work suggests that an alternative adaptive study design (**Figure** **1**), whereby, depending on the prespecified criteria of a study, it is possible that, if the D‐R relationship is sufficiently elucidated after the first or second cohort, the study could be stopped early, therefore reducing the number of subjects overall. However, if the data are highly variable or if the D‐R relationship is not clearly defined, continuing the 2‐2‐4 design for the second and third cohorts (with 3 dose levels in each cohort) could provide a greater number of doses overall to better inform the final model developed from the IBSM study. This retrospective analysis only assessed the model from the first cohort, but, in prospective studies and future confirmatory analyses, subsequent cohorts could use a range of doses until the D‐R relationship is adequately described.

Logistically speaking, the design of an adaptive trial presents inherent difficulties, such as the need for a flexible protocol, time to prepare datasets and perform cohort‐by‐cohort analyses, the assignment of next cohort doses, and a flexible dosage form, which can allow a broad range of doses to be administered. Clinicians, pharmacists, and staff may also require additional training to become familiar with the adaptive protocols and establishing standard operating procedures for dosing regimens and dose administration. In this work, the first‐in‐human data were not available to inform the population PK model early on. However, if first‐in‐human SAD data and preclinical data are available, a population PK or a physiologically‐based pharmacokinetic model could also be developed to inform the first cohort. Additionally, in this analysis, the true distributions of subject weights from the published study as well as the true observed baseline parasite counts from the phase II study were used to simulate values that were included in the analysis. For future analyses and for the workflow outlined in **Figure** **1**, it is recommended that a population database of baseline parasite counts and other covariates (e.g., parasite species, geographical location, weight, and sex) is created, which could be used for future M&S work.

If an IBSM study uses the 2‐2‐4 adaptive design and integrated M&S, it is possible that a useful D‐R relationship, similar to the one which would be obtained from a phase II trial, could be achieved. This work used an empirical PD model to represent parasitemia data during this retrospective M&S analysis. The 2‐2‐4 dataset was comprised of 8, and whereas the Gompertz‐like growth function fit the data of this cohort well, the PD model used in this analysis may not be the most accurate representation of all controlled human malaria infection data. Future work could investigate and compare more mechanistic models with larger datasets as well as investigate the identifiability of model parameters. Ideally, a mechanistic function which governs the prediction of recrudescence could improve the predictions. The most informative PK/PD model would have systemic PD parameters, which would stay constant across models of drug products.

The three dose levels used in this analysis were the dose levels used in the IBSM study already conducted; in practice, these initial three doses would be chosen from preclinical D‐R data and knowledge on safety and PK in healthy volunteers from a phase I SAD study. The number of subjects per cohort was constructed as 2‐2‐4 with a greater number of subjects assigned to the highest dose level, to allow for the highest dose to have the greatest information contributing to the dataset. The scope of this proof‐of‐concept work was not to power each dose level within the cohorts, but to investigate if a multiple‐dose cohort could be useful to build a D‐R relationship. Future work could include a formal statistical analysis for powering each dose level. The first‐in‐human study found doses up to 1,200 mg to be safe, which allowed for a broader range of possible doses to be considered in the 2‐2‐4 cohort.^14^ As a proof of concept, we have gone through this exercise with one random draw of the 2‐2‐4 cohort and demonstrated the PK/PD model outcomes and selection of doses for the next cohort. However, this single‐sampling selection of subjects is a limitation which needs to be addressed in subsequent work. Next steps for an evaluation of this study design could include Markov Chain Monte Carlo simulations of the pilot cohort design using an accepted model for a specific drug. Recrudescence rates at various dose levels would be prespecified and these simulations would be fit with NONMEM. Criteria to select target doses for the subsequent simulated study cohorts would be used and the results of the subsequent cohorts would be simulated; error rates for the conclusions of the simulations based on the specified parameters would then be calculated to evaluate the efficiency and accuracy of results produced from the workflow. The results of this analysis need to be confirmed with additional M&S exercises before recommending a defined workflow, however, this work provides a preliminary workflow, which can be explored for generalization with the end goal of using multiple‐dose in cohort adaptive trial designs in IBSM studies to expedite antimalarial drug development.

This work represents the first retrospective analysis of IBSM study data with the objective to optimize future study design for the development of antimalarial drugs. These analyses support the ongoing efforts to implement PK/PD M&S as part of antimalarial drug development to enable model‐driven decision making. The 2‐2‐4 adaptive design has the potential to accelerate the dose‐selection process through the use of initial cohort data to estimate a useful D‐R relationship. Further exploration of these alternative adaptive study designs is warranted to make more efficient use of volunteer infection studies by reducing the number of subjects and increasing the confidence in a D‐R relationship created from study data, which can then be leveraged for use in guiding doses for selection of combinations.

## Funding

Cognigen Corporation received financial support from the Bill & Melinda Gates Foundation to perform these analyses. Medicines for Malaria Venture funded the studies that produced the data for the analyses. J.J.M. and N.G. are employees of the Medicines for Malaria Venture.

## Conflict of Interest

Cognigen Corporation received financial support from the Bill & Melinda Gates Foundation to perform these analyses. J.S.O. and T.H.G. are employees of Cognigen Corporation. All other authors declared no competing interests for this work.

## Author Contributions

K.A.A., J.S.O., J.M., D.W., N.G., T.H.G., and J.J.M. wrote the manuscript. K.A.A., J.S.O., J.M., D.W., N.G., T.H.G., and J.J.M. designed the research. K.A.A. performed the research. K.A.A. analyzed the data.

## Supporting information

Supplementary Material
