# Supplementary material for: Retrospective Analysis Using Pharmacokinetic/Pharmacodynamic Modeling and Simulation Offers Improvements in Efficiency of the Design of Volunteer Infection Studies for Antimalarial Drug Development
Source: Clin Transl Sci. 2020 Dec 16;14(2):712–9. doi: 10.1111/cts.12934 (PMC7993277; doi:10.1111/cts.12934)
Supplement: Supplementary file 1 — Supplementary Material [file CTS-14-712-s001.docx]

**SUPPLEMENTAL**

The pharmacodynamic model used follows a general growth law assuming a Gompertz form which imparts a degree of deceleration of growth as the population gets more dense. The Gompertz form has a deceleration value which was fixed to 0.1 and is unitless. This model has a parameter, α, which is the maximum parasite count achievable in the human host (parasites/mL); as the parasite counts increase towards α, saturation is reached. This asymptote for maximum parasite growth was set to 1x10^6^ parasites/mL, which is reasonable given that the maximum parasite count observed in the IBSM study was 1x10^5^ parasites/mL.


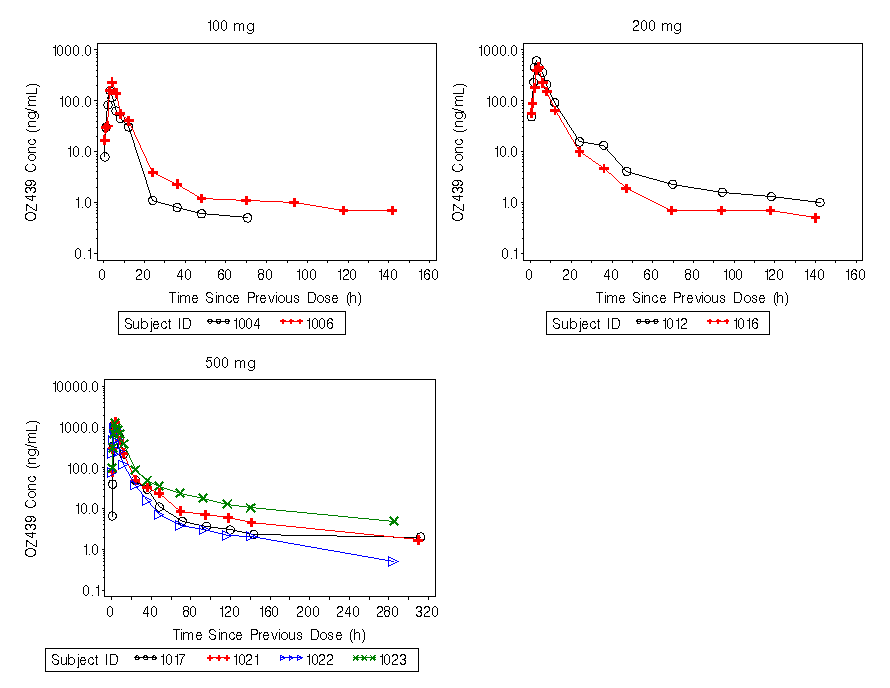


Figure S1 OZ439 pharmacokinetic profiles for 2-2-4 cohort. Conc, concentration.


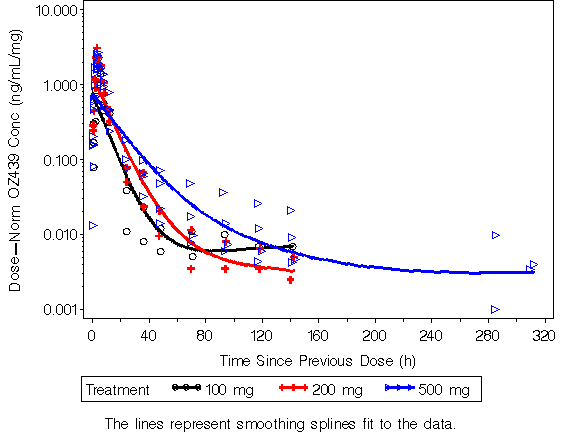


**Figure S2** Dose-normalized OZ439 concentrations for all three cohorts of the 2-2-4 cohort. Conc, concentration; Norm, normalized.


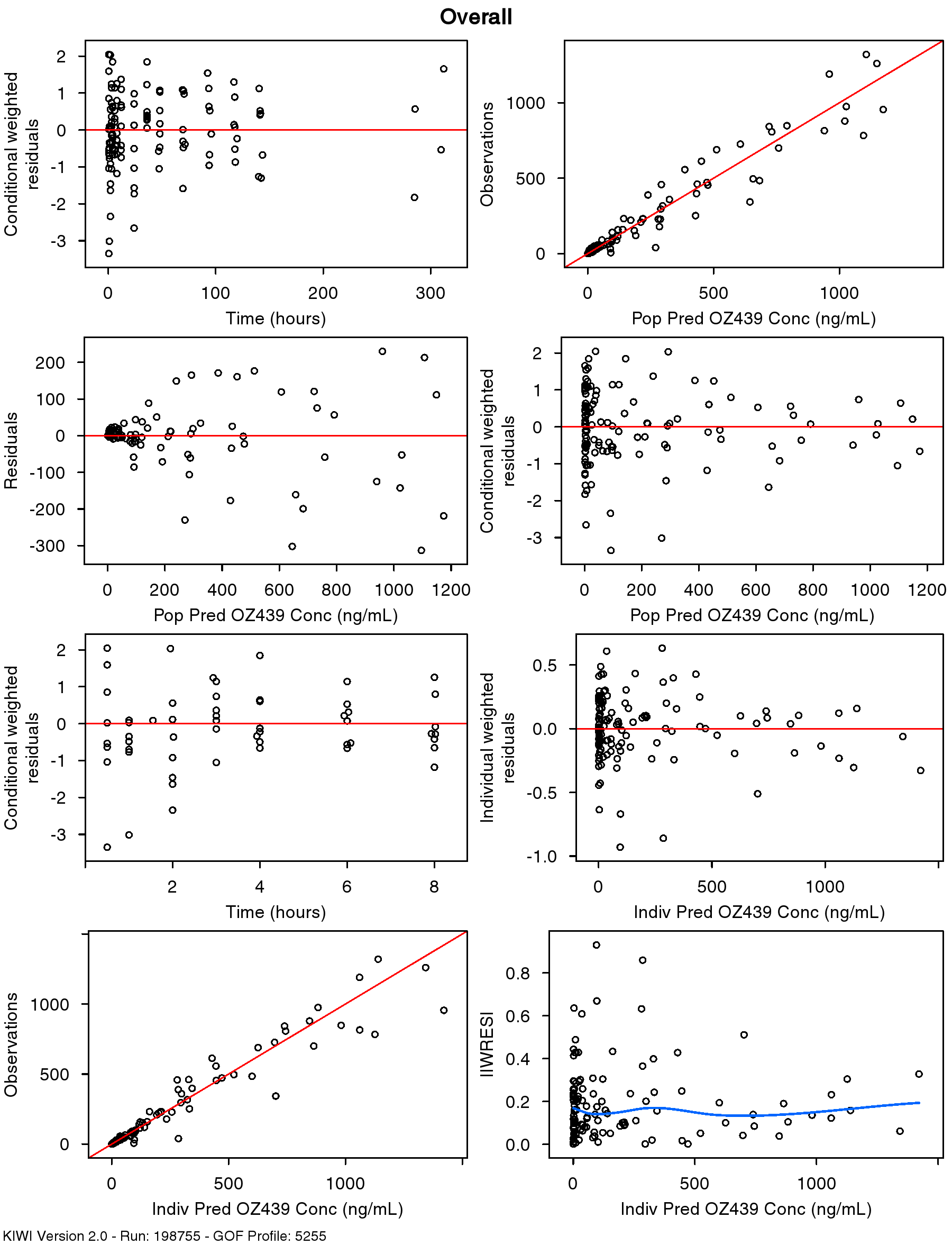


**Figure S3** Goodness-of-fit diagnostic plots for final pharmacokinetic model. |IWRES|, absolute value of the individual weighted residuals.


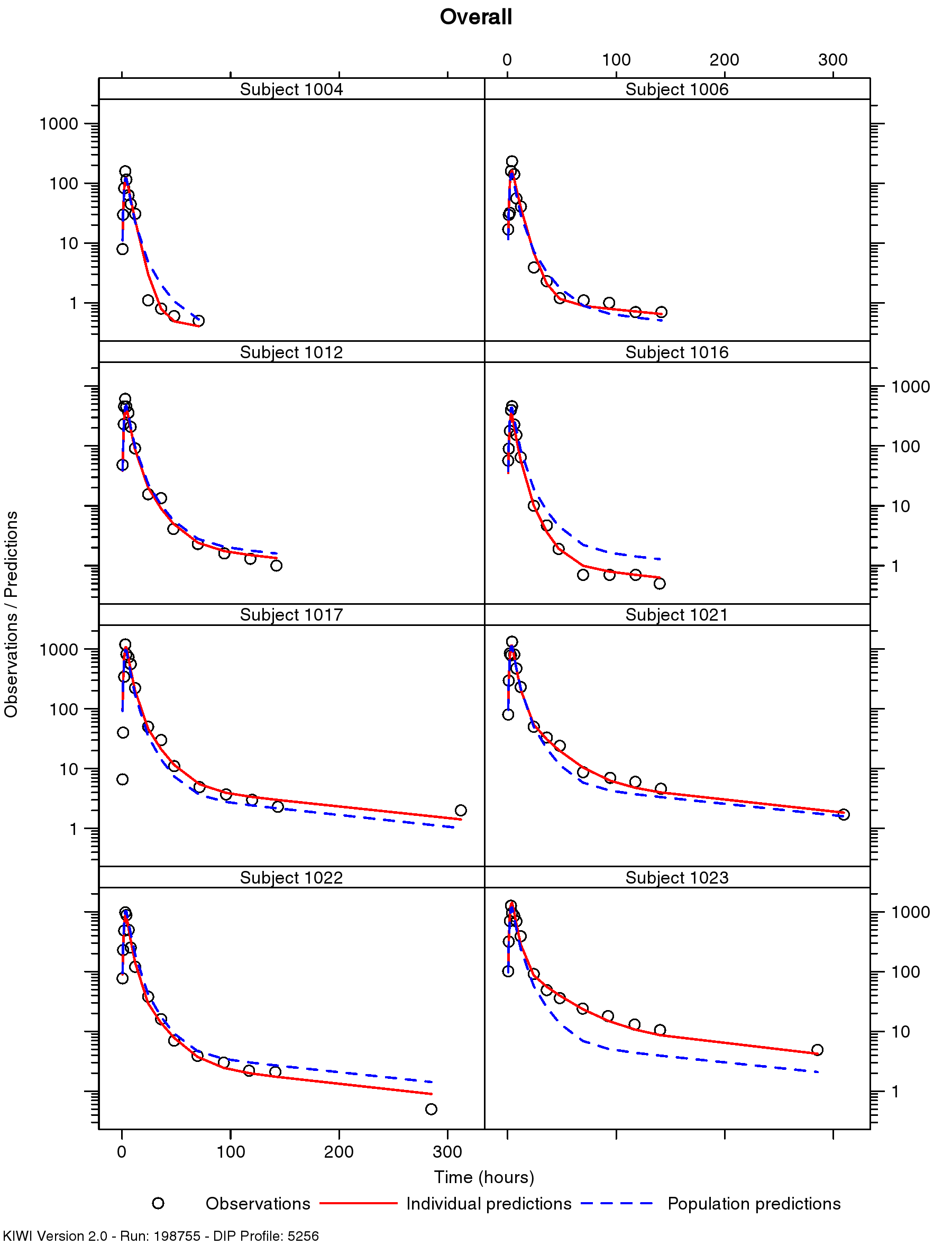


**Figure S4** Observed OZ439 pharmacokinetic profiles overlaid with population and individual model predictions (2-2-4 cohort).


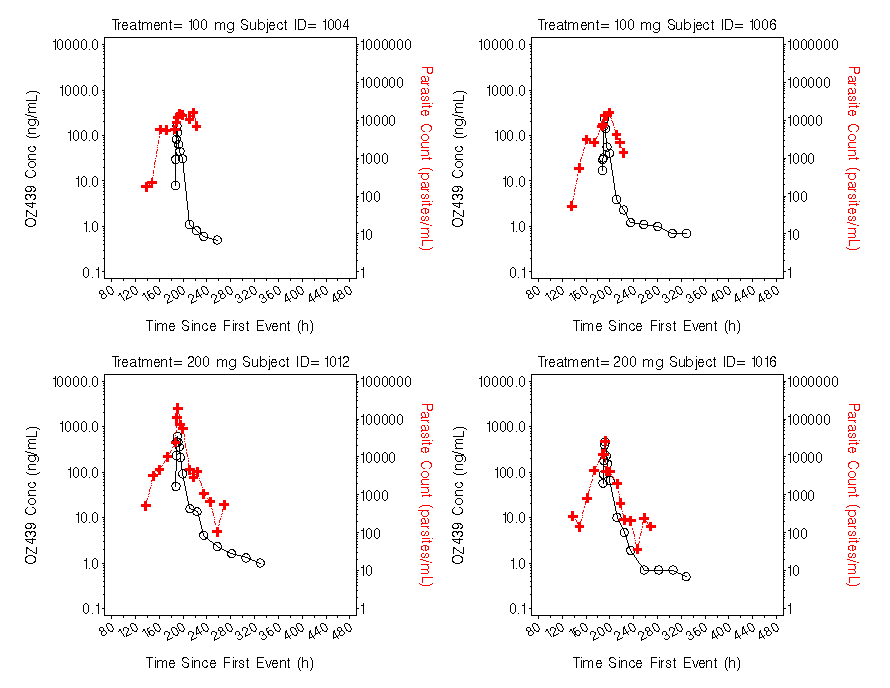


**Figure S5a** Overlay of observed pharmacokinetic and pharmacodynamic profiles (2-2-4 cohort). Conc, concentration; ID, identification number.


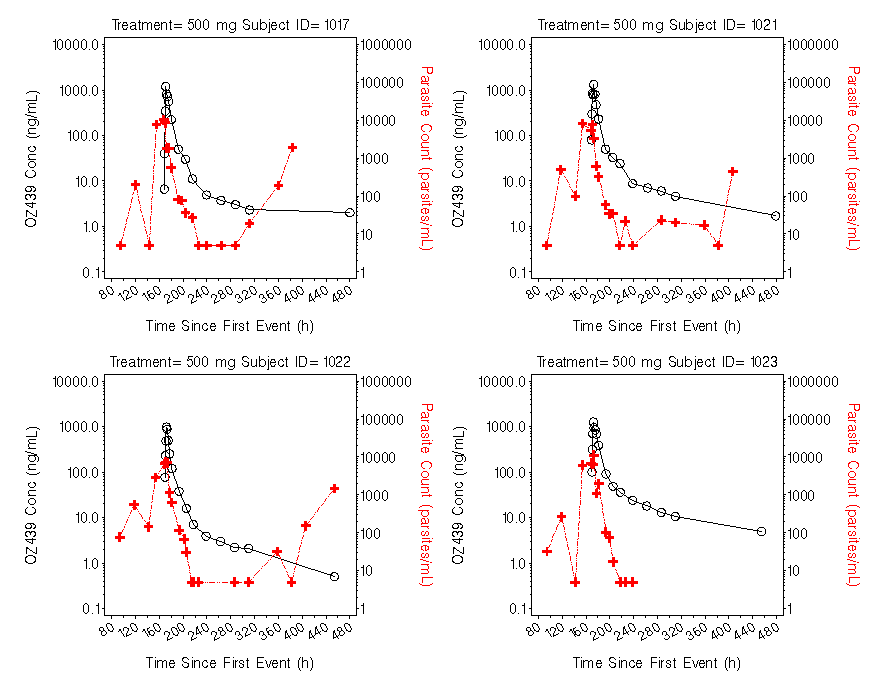


**Figure S5b** Overlay of pharmacokinetic and pharmacodynamic profiles (2-2-4 cohort). Conc, concentration; ID, identification number.


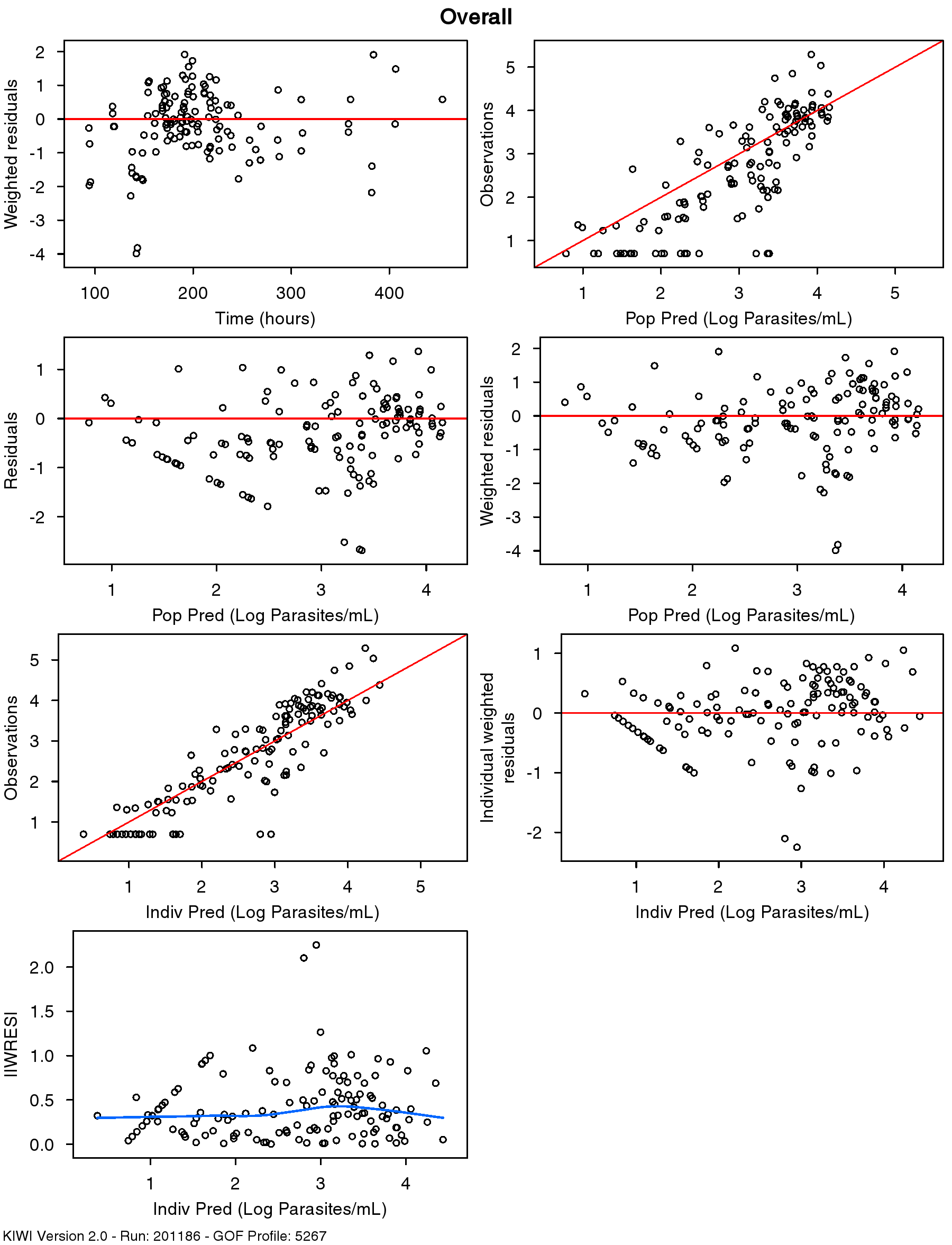


**Figure S6** Goodness-of-fit diagnostic plots from OZ439 pharmacokinetic/pharmacodynamic model developed from 2-2-4 cohort. |IWRES|, absolute value of the individual weighted residuals.


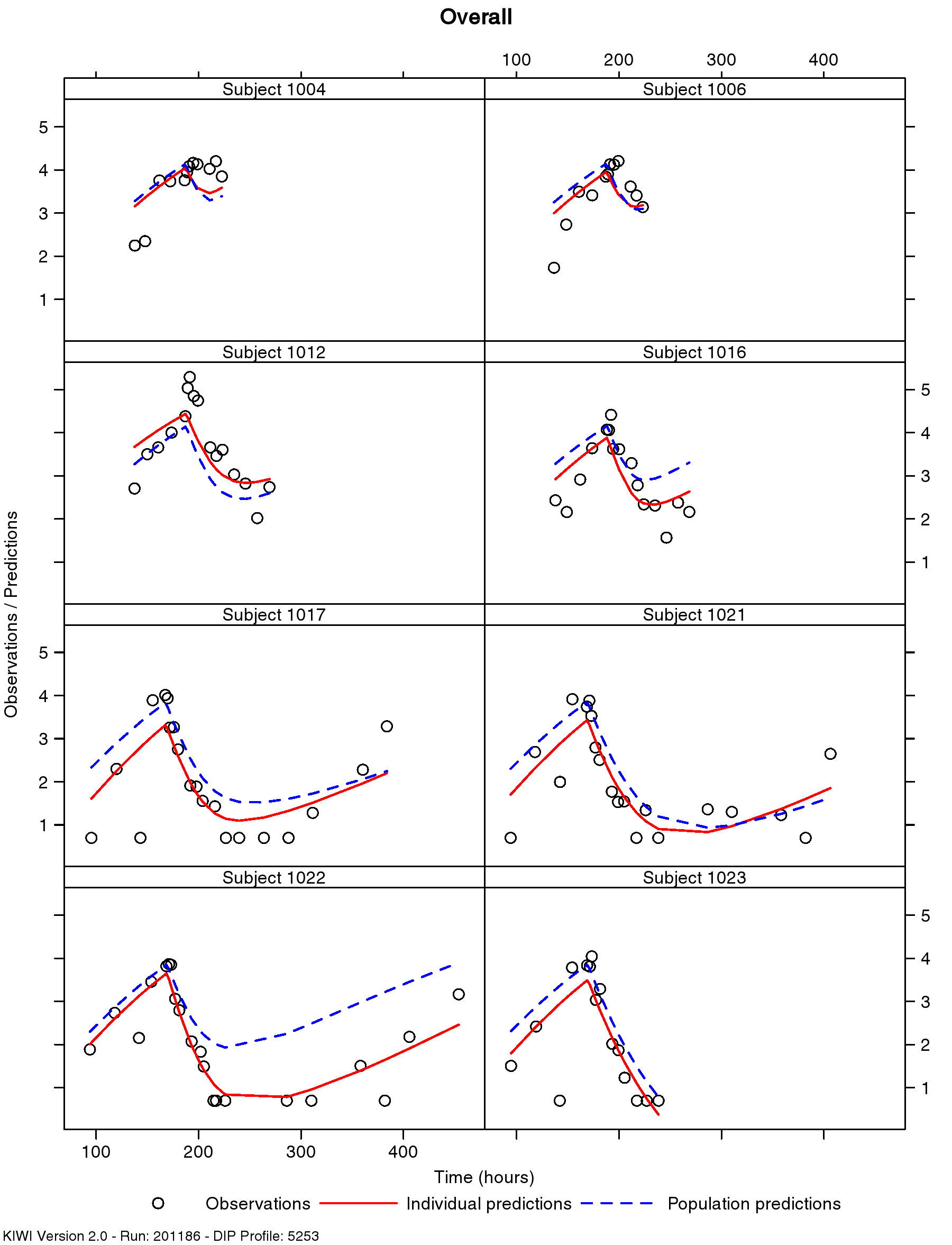


**Figure S7** Observed OZ439 pharmacodynamic profiles overlaid with population and individual model predictions (2-2-4 cohort).


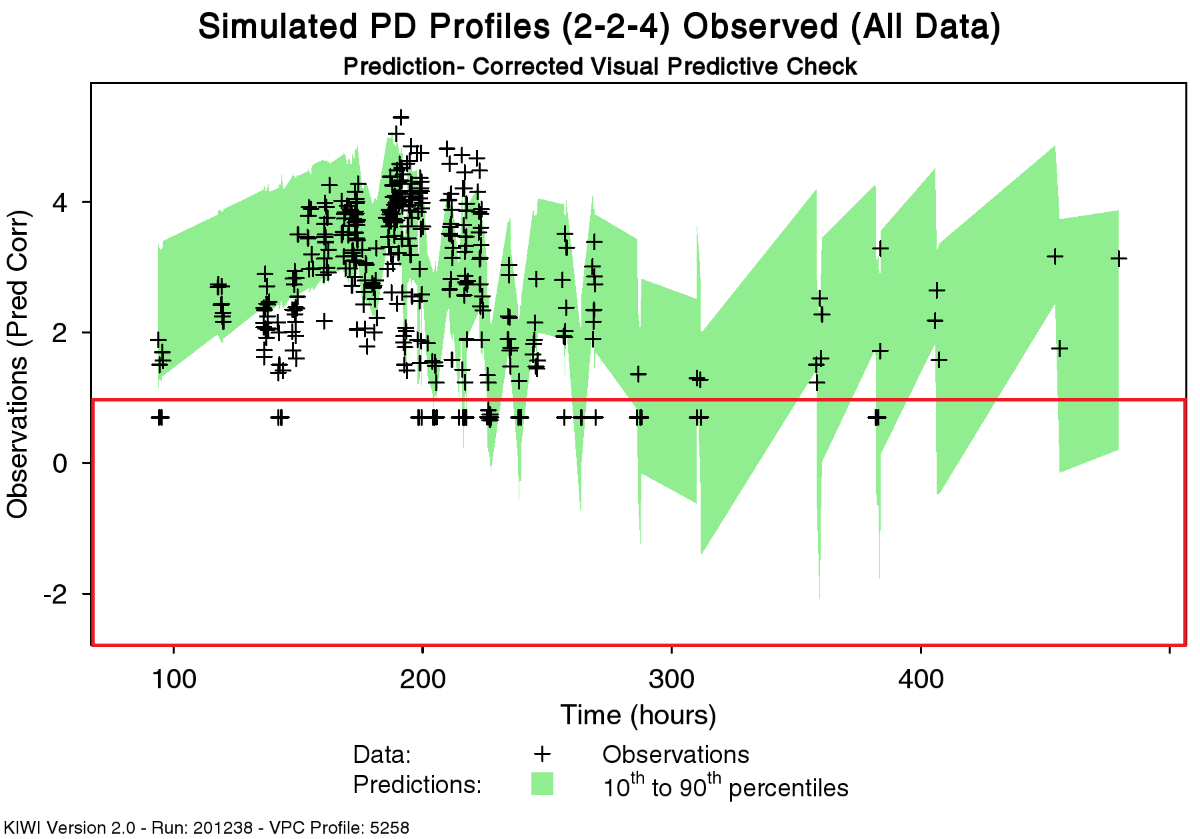


**Figure S8** Prediction-corrected visual predictive check of model developed from 2-2-4 cohort with observed data from the full IBSM study overlaid. IBSM, controlled human malaria infection; PD, pharmacodynamic; Pred Corr, prediction-corrected. The red box encompasses data below the limit of quantification (10 parasites/mL).

| **Table S1 Demographic information (2-2-4 cohort)** | | | | | |
| --- | --- | --- | --- | --- | --- |
| **Subject characteristic** | **Statistic** | **100 mg** | **200 mg** | **500 mg** | **Overall** |
| Age (y) | Mean (SD) | 25.0 (2.8) | 25.0 (2.8) | 26.3 (1.5) | 25.6 (1.9) |
|  | Median | 25.0 | 25.0 | 26.0 | 26.0 |
|  | Min, Max | 23, 27 | 23, 27 | 25, 28 | 23, 28 |
|  | n | 2 | 2 | 4 | 8 |
| Baseline body weight (kg) | Mean  (SD) | 67.40 (16.83) | 63.90  (7.50) | 73.83 (13.55) | 69.74 (12.17) |
|  | Median | 67.40 | 63.90 | 72.50 | 68.10 |
|  | Min, Max | 55.5, 79.3 | 58.6, 69.2 | 59.6, 90.7 | 55.5, 90.7 |
|  | n | 2 | 2 | 4 | 8 |
| Baseline height (cm) | Mean (SD) | 173.5 (16.3) | 174.0 (1.4) | 177.8 (11.2) | 175.8 (9.8) |
|  | Median | 173.5 | 174.0 | 177.0 | 175.0 |
|  | Min, Max | 162, 185 | 173, 175 | 165, 192 | 162, 192 |
|  | n | 2 | 2 | 4 | 8 |
| Sex, n (%) | Male | 1 (50.0) | 1 (50.0) | 2 (50.0) | 4 (50.0) |
|  | Female | 1 (50.0) | 1 (50.0) | 2 (50.0) | 4 (50.0) |
| Max, maximum; Min, minimum; n, number of subjects; SD, standard deviation. | | | | | |

| Table S2 Confidence intervals of estimated pharmacodynamic parameters from 2-2-4 model | | |
| --- | --- | --- |
| Symmetric 95% confidence intervals | | |
| Parameter | 95% confidence interval | |
|  | Lower bound | Upper bound |
| K_g_: first-order growth rate of parasite (1/h) | 0.00491 | 0.0149 |
| EC_50_: concentration of OZ439 at 50% of maximum parasite death (ng/mL) | -1.54 | 18.0 |
| E_max_: maximum parasite death rate from drug (1/h) | 0.0368 | 0.330 |
| Parasite count at baseline | -22.0 | 27.3 |
| IIV on EC_50_ | -1.35 | 1.68 |
| IIV on E_max_ | -0.165 | 0.221 |
| Pharmacodynamic residual variability SD (log unit) | 0.181 | 0.526 |
| IIV, interindividual variability; SD, standard deviation. | | |

| **Table S3 Parameter estimates and standard errors from the 2-2-4 model fit to the full study data** | | | | |
| --- | --- | --- | --- | --- |
| Parameter | Final parameter estimate | | Interindividual variability / residual variability | |
|  | Typical value | %RSE | Magnitude | %RSE |
| K_g_: first-order growth rate of parasite (1/h) | 0.00920 | 7.34 | NE | NA |
| EC_50_: concentration of OZ439 at 50% of maximum parasite death (ng/mL) | 21.2 | 81.2 | 231 %CV | 91.0 |
| Gamma: steepness parameter for concentration response (unitless) | 1.00 | FIXED | NE | NA |
| E_max_: maximum parasite death rate from drug (1/h) | 0.163 | 11.9 | 25.0 %CV | 134 |
| Parasite count at baseline | NA | NA | 181 %CV | 65.0 |
| Pharmacokinetic residual variability | 0.0747 | FIXED | 27.3 %CV | NA |
| Pharmacodynamic residual variability SD (log unit) | 0.271 | 9.60 | 0.520 SD | NA |
| Minimum value of the objective function = 2026.985 | | | | |
| %CV, coefficient of variation expressed as a percent; NA, not applicable; NE, not estimated; %RSE, relative standard error expressed as a percent; SD, standard deviation.  The eta shrinkage was < 36% for K_g_, EC_50_, and E_max_.  The condition number (ratio of the largest to smallest eigenvalue) was 33.8. | | | | |

| Table S4 Confidence intervals of estimated pharmacodynamic parameters from 2-2-4 model fit to all study data | | |
| --- | --- | --- |
| Symmetric 95% confidence intervals | | |
| Parameter | 95% confidence interval | |
|  | Lower bound | Upper bound |
| K_g_: first-order growth rate of parasite (1/h) | 0.00787 | 0.0105 |
| EC_50_: concentration of OZ439 at 50% of maximum parasite death (ng/mL) | -12.6 | 55.0 |
| E_max_: maximum parasite death rate from drug (1/h) | 0.125 | 0.201 |
| Parasite count at baseline | -0.903 | 7.48 |
| IIV on EC_50_ | -4.20 | 14.9 |
| IIV on E_max_ | -0.101 | 0.227 |
| Pharmacodynamic residual variability SD (log unit) | 0.220 | 0.321 |
| IIV, interindividual variability; SD, standard deviation. | | |
